# Oxidative Stress Biomarkers and Their Association with Interleukin-6 in Patients with Obstructive Sleep Apnea: A Cross-Sectional Study

**DOI:** 10.3390/ijms27156879

**Published:** 2026-08-01

**Authors:** Crina Veronica Zinveliu (Bercian), Dan Claudiu Măgureanu, Raluca Maria Pop, Adela-Viviana Sitar-Tăut, Angela Cozma, Olga Hilda Orășan, Roxana Liana Lucaciu, Adriana Corina Hangan, Lucia Maria Procopciuc

**Affiliations:** 1 Department of Cardiology, “Dr. Constantin Papilian” Emergency Military Hospital, 400132 Cluj-Napoca, Romania; crina.bercian@gmail.com; 2 Doctoral School, “Iuliu Hațieganu” University of Medicine and Pharmacy, 400347 Cluj-Napoca, Romania; 3 Pharmacology, Toxicology and Clinical Pharmacology, Department of Morphofunctional Sciences, Faculty of Medicine, “Iuliu Hațieganu” University of Medicine and Pharmacy, 400012 Cluj-Napoca, Romania; raluca.pop@umfcluj.ro; 4 4th Department of Internal Medicine, Faculty of Medicine, “Iuliu Hațieganu” University of Medicine and Pharmacy, 400012 Cluj-Napoca, Romania; adela.sitar@umfcluj.ro (A.-V.S.-T.); angelacozma@umfcluj.ro (A.C.); hilda.orasan@umfcluj.ro (O.H.O.); 5 Department of Pharmaceutical Biochemistry and Clinical Laboratory, Faculty of Pharmacy, “Iuliu-Hațieganu” University of Medicine and Pharmacy, 400012 Cluj-Napoca, Romania; liana.lucaciu@umfcluj.ro; 6 Department of Inorganic Chemistry, Faculty of Pharmacy, “Iuliu-Hațieganu” University of Medicine and Pharmacy, 400012 Cluj-Napoca, Romania; adriana.hangan@umfcluj.ro; 7 Department of Medical Biochemistry, Faculty of Medicine, “Iuliu Hațieganu” University of Medicine and Pharmacy, 400012 Cluj-Napoca, Romania; lprocopciuc@umfcluj.ro

**Keywords:** obstructive sleep apnea, oxidative stress, malondialdehyde, oxidative stress index, interleukin-6, inflammation, nitrosative stress, 3-nitrotyrosine, echocardiography

## Abstract

Oxidative stress and systemic inflammation are considered key mechanisms linking obstructive sleep apnea (OSA) to cardiovascular disease. This study aimed to investigate the relationships among oxidative stress, nitrosative stress, inflammatory biomarkers, and echocardiographic alterations in OSA. This cross-sectional observational study included 72 adults with OSA, classified according to disease severity as mild, moderate, or severe. Clinical characteristics and echocardiographic parameters were assessed. Oxidative stress biomarkers—malondialdehyde (MDA), total oxidant status (TOS), total antioxidant capacity (TAC), nitric oxide (NO), oxidative stress index (OSI), and paraoxonase-1 (PON1); the nitrosative stress marker 3-nitrotyrosine (3-NT), inflammatory biomarkers—interleukin-6 (IL-6) and tumor necrosis factor-alpha (TNF-α); and N-terminal pro-B-type natriuretic peptide (NT-proBNP)—were determined using spectrophotometric and ELISA methods. Correlation and regression analyses were performed to evaluate associations between oxidative stress and inflammation. No significant differences in echocardiographic parameters, oxidative stress biomarkers, inflammatory markers, or NT-proBNP concentrations were observed across OSA severity categories (all *p* > 0.05). Patients with diabetes mellitus exhibited larger left and right atrial diameters and higher pulmonary artery systolic pressure values compared with non-diabetic patients (all *p* < 0.05). Significant positive correlations were identified between IL-6 and MDA (ρ = 0.364, *p* = 0.002), TOS (ρ = 0.259, *p* = 0.029), and OSI (ρ = 0.257, *p* = 0.030). The nitrosative stress marker 3-NT was also positively correlated with MDA, TOS, and OSI (all *p* < 0.05). In multivariable regression analyses adjusted for age, body mass index, and diabetes mellitus, MDA remained independently associated with IL-6 concentrations (B = 1.776, 95% CI 0.110–3.442, standardized β = 0.271, *p* = 0.037). Similarly, OSI remained independently associated with IL-6 (B = 0.043, 95% CI 0.003–0.083, standardized β = 0.277, *p* = 0.034). OSA severity was not associated with significant differences in oxidative stress, inflammatory, or echocardiographic parameters. However, oxidative stress biomarkers were strongly interrelated and remained significantly associated with IL-6 concentrations, suggesting a potential link between oxidative stress and systemic inflammation in patients with OSA. These findings support further investigation of oxidative stress pathways as biomarkers of disease-related biological activity in OSA.

## 1. Introduction

Obstructive sleep apnea (OSA) is one of the most prevalent sleep-related breathing disorders and is characterized by recurrent episodes of upper airway obstruction during sleep, resulting in intermittent hypoxia, sleep fragmentation, intrathoracic pressure fluctuations, and sympathetic activation [1]. Beyond its effects on sleep quality, OSA is increasingly recognized as a systemic disorder associated with substantial cardiovascular morbidity and mortality. Among the mechanisms implicated in OSA-related cardiovascular injury, oxidative stress and systemic inflammation are considered key pathophysiological mediators [2]. Recurrent cycles of hypoxia and reoxygenation promote excessive generation of reactive oxygen species (ROS), leading to disruption of the pro-oxidant/antioxidant balance, lipid peroxidation, mitochondrial dysfunction, endothelial injury, and activation of inflammatory signaling pathways [2,3,4,5,6]. Intermittent hypoxia stimulates redox-sensitive transcription factors, including nuclear factor-kappa B (NF-κB), resulting in increased production of pro-inflammatory cytokines and vascular inflammation [2,5,7]. Collectively, these processes contribute to endothelial dysfunction, atherosclerosis, vascular remodeling, and adverse cardiovascular outcomes [2,5,8].

Several circulating biomarkers have been proposed to characterize oxidative stress and antioxidant defense mechanisms in OSA. Malondialdehyde (MDA), a byproduct of lipid peroxidation, is one of the most extensively investigated markers of oxidative damage and has consistently been reported to be elevated in patients with OSA [9,10]. Total oxidant status (TOS), total antioxidant capacity (TAC), and the oxidative stress index (OSI) provide complementary information regarding the balance between pro-oxidant and antioxidant pathways, whereas reduced nitric oxide (NO) bioavailability reflects endothelial dysfunction associated with intermittent hypoxia and oxidative stress [11,12,13]. In addition, paraoxonase-1 (PON1), an antioxidant enzyme associated with high-density lipoproteins, contributes to protection against lipid oxidation and vascular damage, while 3-nitrotyrosine (3-NT) is a stable marker of protein nitration generated by reactive nitrogen species and peroxynitrite-mediated injury [13,14,15]. Increased nitrotyrosine expression in vascular tissues of patients with OSA further supports the presence of nitrosative stress alongside oxidative stress [13,14,15,16].

Inflammation represents another hallmark of OSA-related cardiovascular injury. Interleukin-6 (IL-6) and tumor necrosis factor-alpha (TNF-α) have received particular attention because of their roles in endothelial dysfunction, vascular remodeling, and atherosclerotic disease progression [17,18]. Elevated circulating concentrations of these cytokines have been reported in patients with OSA and are associated with increased cardiovascular risk [7,17,18]. Although oxidative stress and inflammation are recognized as closely interconnected processes in OSA, their interactions remain incompletely understood, particularly in the presence of common cardiometabolic comorbidities such as obesity and diabetes mellitus, which may independently influence redox balance and inflammatory activity [2,7,10,19]. In addition to biochemical alterations, OSA has been associated with structural and functional cardiovascular remodeling, including atrial enlargement, pulmonary hypertension, and right ventricular dysfunction [2,20,21,22]. Oxidative stress, nitrosative stress, chronic inflammation, and intermittent hypoxia are increasingly recognized as important contributors to these cardiovascular abnormalities [2,16,22]. However, it remains unclear whether these biological alterations are primarily related to OSA severity itself or are substantially influenced by accompanying cardiometabolic conditions. Moreover, data regarding the relationships among oxidative stress biomarkers, nitrosative stress markers, inflammatory mediators, and echocardiographic abnormalities remain limited. A more integrated evaluation of these pathways may improve understanding of the mechanisms linking OSA to cardiovascular disease and help identify biologically relevant markers beyond conventional disease severity classification [2,7].

Therefore, the aim of the present study was to investigate the relationships between oxidative stress, nitrosative stress, inflammatory biomarkers, and echocardiographic parameters in patients with obstructive sleep apnea syndrome. In addition, we sought to explore the association between oxidative stress biomarkers and systemic inflammatory activity, as reflected by circulating IL-6 concentrations.

## 2. Results

### 2.1. Study Population Characteristics

A total of 72 patients with obstructive sleep apnea syndrome (OSA) were included in the study. The median age of the cohort was 59 years (IQR: 49–64), and most participants were male (73.6%). The study population was characterized by a high prevalence of obesity, with a median body mass index (BMI) of 34.2 kg/m^2^ (IQR: 31.3–38.0).

Regarding OSA severity, 15 patients (20.8%) had mild disease, 19 (26.4%) had moderate disease, and 38 (52.8%) had severe disease, indicating that nearly four-fifths of the cohort presented with moderate-to-severe OSA. Hypertension was the most frequent comorbidity (93.1%), followed by chronic pulmonary disease (34.7%), ischemic heart disease (27.8%), and diabetes mellitus (22.2%).

Baseline demographic, clinical, echocardiographic, oxidative stress, inflammatory, and cardiac biomarker characteristics are summarized in Table 1.

### 2.2. Relationship Between OSA Severity and Clinical, Echocardiographic, and Biochemical Parameters

Comparative analyses were performed according to OSA severity categories (mild, moderate, and severe), and the results are summarized in Table 2.

No significant differences were observed among severity groups regarding age, body mass index, renal function, inflammatory status, or the prevalence of major comorbidities, including diabetes mellitus, ischemic heart disease, and chronic pulmonary disease (all *p* > 0.05). Similarly, echocardiographic measurements, including left atrial diameter, right atrial diameter, right ventricular diameter, TAPSE, and pulmonary artery systolic pressure, did not differ significantly across OSA severity categories. Oxidative stress biomarkers (MDA, TOS, TAC, NO, OSI, PON1, and 3-nitrotyrosine), inflammatory markers (TNF-α and IL-6), and NT-proBNP concentrations also showed no significant differences among severity groups (all *p* > 0.05). Although patients with severe OSA tended to exhibit higher median TOS and OSI values compared with those with mild or moderate disease, these differences did not reach statistical significance (Table 2). Overall, OSA severity was not associated with significant differences in clinical characteristics, echocardiographic parameters, oxidative stress biomarkers, inflammatory markers, or NT-proBNP concentrations in the present cohort.

### 2.3. Influence of Cardiometabolic Comorbidities

Given the high prevalence of diabetes mellitus within the study cohort, additional analyses were performed to evaluate its potential impact on echocardiographic, oxidative stress, and inflammatory parameters (Table 3).

Patients with diabetes mellitus exhibited significantly larger left atrial diameter (31 [29–33.8] vs. 30 [28–30] mm, *p* = 0.041) and right atrial diameter (17.5 [17–18] vs. 17 [16–18] mm, *p* = 0.010) compared with non-diabetic patients. In addition, pulmonary artery systolic pressure was significantly higher in diabetic individuals (35 [32–37] vs. 32 [20–36] mmHg, *p* = 0.026). Regarding oxidative stress biomarkers, diabetic patients showed numerically higher TOS and OSI values than non-diabetic patients; however, these differences did not reach statistical significance (TOS: *p* = 0.082; OSI: *p* = 0.078). MDA concentrations were comparable between groups (*p* = 0.461). Overall, diabetes mellitus was associated with markers of cardiac structural remodeling and higher pulmonary pressures, whereas oxidative stress biomarkers demonstrated only non-significant trends toward higher values in diabetic patients.

### 2.4. Association Between Oxidative Stress, Nitrosative Stress, and Inflammatory Biomarkers

To investigate the relationships among oxidative stress, nitrosative stress, inflammation, and cardiac biomarkers, Spearman correlation analyses were performed (Table 4).

Strong positive correlations were identified among oxidative stress markers. MDA was strongly correlated with both TOS (ρ = 0.752, *p* < 0.001) and OSI (ρ = 0.740, *p* < 0.001), while TOS and OSI demonstrated an almost perfect positive correlation (ρ = 0.999, *p* < 0.001). Significant associations were also observed between oxidative stress and inflammatory activity. IL-6 levels correlated positively with MDA (ρ = 0.364, *p* = 0.002), TOS (ρ = 0.259, *p* = 0.029), and OSI (ρ = 0.257, *p* = 0.030). In addition, the nitrosative stress marker 3-nitrotyrosine was positively associated with MDA (ρ = 0.305, *p* = 0.010), TOS (ρ = 0.375, *p* = 0.001), and OSI (ρ = 0.374, *p* = 0.001). No significant correlations were observed between oxidative stress biomarkers and TNF-α, CRP, NT-proBNP, or PON1 concentrations. Likewise, IL-6 was not significantly associated with 3-nitrotyrosine, TNF-α, CRP, NT-proBNP, or PON1.

### 2.5. Association of Oxidative Stress with Circulating IL-6 Levels

Because IL-6 demonstrated significant correlations with several oxidative stress biomarkers, linear regression analyses were performed to further investigate these relationships (Table 5).

In univariate analyses, both MDA and OSI were significantly associated with IL-6 concentrations. MDA explained 5.7% of IL-6 variability (R^2^ = 0.057, β = 0.238, *p* = 0.045), whereas OSI explained 7.4% of IL-6 variability (R^2^ = 0.074, β = 0.272, *p* = 0.022). To evaluate whether these associations persisted after accounting for potential clinical confounders, multivariable regression models adjusted for age, body mass index, and diabetes mellitus were constructed. In the adjusted models, MDA remained significantly associated with IL-6 concentrations (B = 1.776, 95% CI 0.110–3.442, standardized β = 0.271, *p* = 0.037). Similarly, OSI remained significantly associated with IL-6 levels (B = 0.043, 95% CI 0.003–0.083, standardized β = 0.277, *p* = 0.034). In contrast, age, body mass index, and diabetes mellitus were not significantly associated with IL-6 in either model.

## 3. Discussion

The present study explored the interplay between oxidative stress, nitrosative stress, systemic inflammation, and cardiovascular remodeling in patients with OSA. The principal findings were that conventional OSA severity categories were not associated with significant differences in oxidative stress, inflammatory, or echocardiographic parameters; diabetes mellitus was associated with markers of cardiac remodeling; and oxidative stress biomarkers demonstrated significant associations with both IL-6 and 3-nitrotyrosine. Importantly, MDA and OSI remained independently associated with IL-6 concentrations after adjustment for age, body mass index, and diabetes mellitus.

Oxidative stress is a key pathophysiological mechanism linking OSA with cardiovascular disease through intermittent hypoxia, reactive oxygen species generation, endothelial dysfunction, and vascular injury [1,2,13,23,24]. Consistent with these mechanisms, several studies and meta-analyses have demonstrated increased circulating concentrations of oxidative stress biomarkers in patients with OSA, including malondialdehyde (MDA), total oxidant capacity, and other markers of lipid peroxidation [9,11]. Furthermore, positive associations between oxidative stress biomarkers and OSA severity have been reported, with higher levels generally observed in patients with more severe disease [10,25]. In contrast, we observed no significant differences in oxidative stress biomarkers across conventional OSA severity categories. This discrepancy may reflect the multifactorial nature of oxidative stress in OSA, methodological heterogeneity among studies, and the limited ability of the apnea-hypopnea index alone to capture the cumulative biological burden of intermittent hypoxia. Increasing evidence suggests that physiological measures such as the oxygen desaturation index, hypoxic burden, and the duration of nocturnal hypoxemia may better reflect the mechanisms driving oxidative stress than AHI alone [9]. An additional explanation for the absence of significant differences across OSA severity categories may be the high burden of obesity and cardiometabolic comorbidities within the study population. Obesity is characterized by increased oxidative stress, chronic low-grade inflammation, and sympathetic activation resulting from enhanced lipid peroxidation, mitochondrial dysfunction, and dysregulation of antioxidant defense mechanisms [26,27]. Furthermore, obesity and OSA exert synergistic effects on oxidative stress and inflammation through convergent molecular pathways, including NF-κB signaling and cytokine production [7,27]. Similarly, hypertension and diabetes mellitus represent independent sources of oxidative stress through mechanisms involving NADPH oxidase activation, endothelial dysfunction, mitochondrial impairment, and chronic inflammatory signaling [28,29,30,31]. Consequently, the coexistence of these conditions may obscure the specific contribution of OSA severity to circulating oxidative stress biomarkers. Moreover, because all participants had OSA, the present study cannot determine whether the observed biomarker concentrations are specific to OSA itself or are influenced by the high prevalence of obesity and cardiometabolic comorbidities within the study population. Notably, TOS and OSI showed a progressive, albeit non-significant, increase across OSA severity categories. However, the relatively small sample size, particularly the limited number of patients with mild OSA, may have reduced the statistical power to detect significant between-group differences. Therefore, these negative findings should be interpreted with caution and should not be considered evidence of the absence of biological differences. Larger studies with more homogeneous populations are warranted to clarify these associations.One of the most relevant findings of the present study was the significant association between oxidative stress biomarkers and systemic inflammatory activity. IL-6 demonstrated significant positive correlations with MDA, TOS, and OSI, whereas no significant associations were observed for TNF-α, CRP, or NT-proBNP. Furthermore, both MDA and OSI remained significantly associated with IL-6 concentrations after adjustment for age, body mass index, and diabetes mellitus. These findings are consistent with previous evidence indicating that oxidative stress and inflammation represent closely interconnected biological processes in OSA. Intermittent hypoxia promotes excessive production of reactive oxygen species, which activate redox-sensitive signaling pathways, most notably nuclear factor kappa-B (NF-κB), leading to increased transcription of pro-inflammatory cytokines, including IL-6 [2,32,33,34]. Clinical studies have similarly demonstrated concomitant elevations of oxidative stress and inflammatory biomarkers in OSA patients, with positive correlations between markers of lipid peroxidation and circulating inflammatory mediators [35]. Collectively, these observations support the concept that oxidative stress may contribute to the maintenance of a chronic low-grade inflammatory state in OSA, even in the absence of clear associations between conventional disease severity categories and circulating biomarker concentrations.

In addition to the association between oxidative stress and inflammatory activity, significant correlations were identified between 3-nitrotyrosine and multiple oxidative stress biomarkers, including MDA, TOS, and OSI. As a stable product of protein tyrosine nitration, 3-nitrotyrosine is widely regarded as a marker of nitrosative stress and peroxynitrite-mediated cellular injury. Elevated nitrotyrosine expression has previously been demonstrated in vascular and endothelial tissues of patients with OSA, providing evidence of ongoing nitrosative damage in this population [4,13]. The observed associations between 3-nitrotyrosine and oxidative stress biomarkers support the concept that oxidative and nitrosative pathways are closely interconnected in OSA. Under conditions of increased oxidative stress, excessive superoxide production rapidly reacts with nitric oxide to generate peroxynitrite, a highly reactive oxidant implicated in protein nitration, endothelial dysfunction, and vascular injury [29]. Previous studies have shown that chronic intermittent hypoxia induces a nitro-oxidative and pro-inflammatory milieu associated with endothelial dysfunction, hypertension, and other cardiovascular consequences of OSA [2,13,36]. Therefore, the significant correlations observed between 3-nitrotyrosine and oxidative stress biomarkers provide additional evidence that oxidative and nitrosative injury coexist in OSA and may represent complementary mechanisms contributing to cardiovascular risk. The nearly perfect correlation observed between TOS and OSI was expected, as OSI is mathematically derived from TOS and TAC. Therefore, this relationship should be interpreted as a consequence of the calculation of the index rather than as an independent biological finding.

The multivariable regression analyses provided additional support for the relationship between oxidative stress and systemic inflammation in OSA. Although age, obesity, and diabetes mellitus are well-recognized determinants of inflammatory activity, both MDA and OSI remained significantly associated with circulating IL-6 concentrations after adjustment for these factors. Although the regression models explained only a modest proportion of IL-6 variability (adjusted R^2^ approximately 4–5%), the persistence of these associations after multivariable adjustment suggests that oxidative stress is independently associated with systemic inflammation. However, the limited explanatory power of the models indicates that IL-6 concentrations are influenced by numerous additional biological and clinical factors not captured in the present analyses. Therefore, these findings should be interpreted as evidence of an independent statistical association rather than a comprehensive explanation of inflammatory activity or a direct indication of clinical relevance. The biological relevance of this finding is supported by extensive evidence indicating that OSA is characterized by a chronic low-grade inflammatory state driven by intermittent hypoxia and oxidative stress [2]. Reactive oxygen species generated during recurrent hypoxia-reoxygenation cycles activate redox-sensitive signaling pathways, including NF-κB, leading to increased expression of pro-inflammatory cytokines and endothelial activation [2,37,38]. Among these mediators, IL-6 has emerged as one of the most consistently elevated inflammatory biomarkers in OSA. A recent meta-analysis including 63 studies demonstrated significantly higher circulating IL-6 concentrations in patients with OSA compared with healthy controls, further supporting its role as a central marker of OSA-related inflammatory activity [39]. The clinical implications of these observations are particularly important because chronic inflammation is considered a major mediator of cardiovascular injury in OSA. Persistent activation of inflammatory pathways contributes to endothelial dysfunction, vascular remodeling, atherosclerosis, and the development of cardiovascular comorbidities [2,7,40]. Furthermore, accumulating evidence indicates that treatment with continuous positive airway pressure can reduce both oxidative stress and inflammatory mediators, including IL-6, suggesting that these pathways are not merely epiphenomena but potentially modifiable mechanisms involved in disease progression [7,16,38]. Therefore, the observed association between oxidative stress biomarkers and IL-6 provides additional support for the concept that oxidative stress and inflammation represent closely interconnected processes contributing to the cardiovascular burden of OSA.

Another finding of the present study was the association between the diabetes mellitus and modest differences in cardiac structure and hemodynamics. Patients with diabetes exhibited larger left and right atrial dimensions as well as higher pulmonary artery systolic pressure compared with non-diabetic individuals. These observations are consistent with previous evidence indicating that diabetes promotes myocardial remodeling through mechanisms involving oxidative stress, chronic inflammation, advanced glycation end-product accumulation, and activation of profibrotic signaling pathways [41,42,43]. Over time, these processes contribute to myocardial fibrosis, impaired ventricular relaxation, and diastolic dysfunction, ultimately resulting in atrial enlargement and altered cardiac hemodynamics [41,43]. Both diabetes and OSA share common pathophysiological pathways characterized by increased reactive oxygen species production, endothelial dysfunction, and chronic low-grade inflammation [44,45,46]. Consequently, their coexistence may amplify cardiovascular injury through synergistic effects on oxidative stress and tissue remodeling. Although no statistically significant differences were observed for oxidative stress biomarkers, diabetic patients demonstrated numerically higher TOS and OSI values than non-diabetic individuals, supporting the hypothesis that diabetes may increase oxidative stress burden in patients with OSA [42,43,44,45,46,47]. Although these differences reached statistical significance, their absolute magnitude was modest (approximately 1 mm for left atrial diameter, 0.5 mm for right atrial diameter, and 3 mmHg for pulmonary artery systolic pressure). Therefore, their clinical significance should be interpreted with caution, as these values may approach the expected variability of routine echocardiographic measurements. Although diabetes mellitus was included as a covariate in the multivariable regression models, the present study was not sufficiently powered to determine whether diabetes modifies the association between oxidative stress biomarkers and IL-6 concentrations. Future studies with larger cohorts should investigate potential interaction effects between diabetes, oxidative stress, and systemic inflammation in OSA. Rather than indicating clinically relevant structural abnormalities, these findings may reflect subtle remodeling associated with diabetes mellitus in patients with OSA and should be confirmed in larger prospective studies. The clinical implications of these findings deserve consideration. Although OSA severity is traditionally classified according to the apnea-hypopnea index (AHI), increasing evidence suggests that this metric does not fully capture the biological heterogeneity of the disease or accurately predict long-term cardiovascular risk [48,49]. The AHI fails to account for factors such as the depth and duration of oxygen desaturation, hypoxic burden, sleep fragmentation, and individual susceptibility to intermittent hypoxia [48,49,50,51]. These limitations may partly explain why no significant differences in oxidative stress or inflammatory biomarkers were observed across conventional OSA severity categories in the present study. In this context, biomarker-based assessment may provide complementary information regarding the biological consequences of OSA. Emerging evidence indicates that oxidative stress biomarkers reflect key pathophysiological processes involved in OSA, including endothelial dysfunction, systemic inflammation, and cardiovascular remodeling [10,48,52]. Furthermore, several oxidative stress markers correlate with indices of nocturnal hypoxemia and may be useful for monitoring individual responses to therapy [10,52]. The potential clinical relevance of oxidative stress is further supported by studies demonstrating reductions in oxidative and inflammatory biomarkers following continuous positive airway pressure therapy [10,53]. Although antioxidant-based strategies remain investigational [10,52,54], the present findings suggest that oxidative stress biomarkers may contribute to improved risk stratification and a more comprehensive characterization of OSA beyond conventional severity classification.

Several limitations should be acknowledged. First, the study was conducted in a relatively small, single-center convenience sample, with a limited number of patients in the mild OSA subgroup. In addition, no a priori sample size calculation was performed because recruitment was based on all consecutive eligible patients during the predefined study period. Consequently, the study may have been underpowered to detect more subtle differences across OSA severity categories, although several significant associations were identified. Second, the cross-sectional design precludes conclusions regarding causality. Third, the absence of a control group without OSA precluded comparisons between OSA patients and healthy individuals. Fourth, the high prevalence of obesity, hypertension, and other cardiometabolic comorbidities may have influenced biomarker concentrations independently of OSA severity. Moreover, detailed information regarding chronic medication use, including statins, renin-angiotensin system inhibitors, antidiabetic agents, and antioxidant supplements, was not systematically collected. As these therapies may influence oxidative stress and inflammatory biomarkers, residual confounding cannot be excluded. Furthermore, all echocardiographic examinations were performed by the same experienced investigator using a standardized imaging protocol, thereby minimizing interobserver variability. However, formal intraobserver reproducibility was not assessed, and future studies should incorporate reproducibility analyses to further strengthen the reliability of echocardiographic measurements. In addition, the external validity of the present findings should be considered carefully. Participants were recruited from the sleep laboratory of a single tertiary military emergency hospital, and the study population consisted predominantly of men with obesity and a high prevalence of hypertension and other cardiometabolic comorbidities. Consequently, these findings may not be directly generalizable to community-based OSA populations, women, patients without significant cardiovascular comorbidity, or individuals with non-obese OSA phenotypes. Future multicenter studies including more diverse patient populations are warranted to confirm the present observations. Finally, detailed polysomnographic indices reflecting nocturnal hypoxemia burden (e.g., oxygen desaturation metrics, hypoxic burden, time spent below specific oxygen saturation thresholds) were not incorporated into the present analyses, and future studies should investigate whether specific indices of nocturnal hypoxemia are more closely associated with oxidative stress and inflammatory pathways than conventional severity categories alone.

## 4. Materials and Methods

### 4.1. Study Design and Population

This cross-sectional observational study included 72 consecutive adult patients diagnosed with obstructive sleep apnea (OSA). Participants were recruited from the Sleep Laboratory of “Constantin Papilian” Military Clinical Emergency Hospital between the December 2023 and March 2026. The diagnosis of OSA was established using overnight Type 3 portable respiratory monitoring performed with the Nox T3 system (Nox Medical, Reykjavík, Iceland). Respiratory recordings were initially scored automatically using the manufacturer’s software and subsequently reviewed and corrected manually by a certified sleep specialist in accordance with the American Academy of Sleep Medicine (AASM) scoring criteria. OSA severity was classified according to the apnea-hypopnea index (AHI) as mild (5.0–14.9 events/h), moderate (15.0–29.9 events/h), and severe (≥30 events/h), in accordance with the AASM guidelines.

Eligible participants were adults with newly diagnosed OSA who had not received previous treatment for sleep-disordered breathing. Patients were excluded if they had central or complex sleep apnea, previous or ongoing treatment with continuous positive airway pressure (CPAP), noninvasive ventilation, or oral appliance therapy. Additional exclusion criteria included acute infection or recent febrile illness, autoimmune or active inflammatory diseases, active malignancy, and other medical conditions considered likely to influence oxidative stress or inflammatory biomarker concentrations substantially. Demographic characteristics, anthropometric measurements, smoking status, comorbidities, laboratory data, and echocardiographic parameters were recorded for all participants.

During the study period, all consecutive patients referred to the Sleep Laboratory with suspected OSA were assessed for eligibility. The study sample represented a convenience sample consisting of all consecutive eligible patients with complete clinical, laboratory, sleep study, and echocardiographic data available during the predefined recruitment period. Because the study was conducted within a fixed recruitment period, no a priori sample size calculation was performed. Patients meeting any of the predefined exclusion criteria were not enrolled. All included participants completed the overnight sleep study, blood sampling, and transthoracic echocardiographic evaluation according to the study protocol. All study assessments were performed within a predefined time window of 24–48 h. Venous blood samples were collected on the morning immediately following the overnight sleep study after an overnight fast, and transthoracic echocardiography was performed on the same day as blood sampling. No missing data were identified for the variables included in the present analyses; therefore, all 72 enrolled participants were included in the final statistical analyses.

### 4.2. Blood Sample Collection and Processing

Venous blood samples were collected after the overnight type 3 sleep study, in the morning after overnight fasting. Blood was collected into 4 mL serum separator tubes containing clot activator and separation gel. Samples were centrifuged within 30 min of collection, at room temperature for 10 min at 4000 rpm. After serum separation, the samples were aliquoted and stored at −80 °C until further analysis. Because patients were enrolled from December 2023 until March 2026, the storage period varied according to the enrollment date. All laboratory analyses were performed using the assay kits described below, according to the manufacturers’ instructions.

### 4.3. Clinical and Laboratory Assessment

Baseline demographic and clinical data included age, sex, body mass index (BMI), smoking status, hypertension, diabetes mellitus, ischemic heart disease, atrial fibrillation, chronic pulmonary disease, estimated glomerular filtration rate (eGFR), and C-reactive protein (CRP). Venous blood samples were obtained after overnight fasting and processed according to institutional laboratory protocols.

Oxidative stress was evaluated by measuring serum levels of malondialdehyde (MDA), total oxidant status (TOS), total antioxidant capacity (TAC), nitric oxide (NO), and oxidative stress index (OSI). All spectrophotometric determinations were performed using a Jasco V-530 UV–Vis spectrophotometer (JASCO International Co. Ltd., Tokyo, Japan) according to previously described methods [55].

Serum concentrations of paraoxonase-1 (PON1; E-EL-H2298), tumor necrosis factor-α (TNF-α; E-EL-H0109), and interleukin-6 (IL-6; E-EL-H6156) were determined using a commercially available ELISA kit from Elabscience Biotechnology Inc., Houston, TX, USA. N-terminal pro-B-type natriuretic peptide (NT-proBNP; E-EL-H6126) was quantified as a biomarker of myocardial stress. Nitrosative stress was assessed by measuring serum 3-nitrotyrosine (3-NT; abx257155) using a commercially available ELISA kit from Abbexa Ltd., Cambridge, UK. All parameters were analysed as single determinations. The manufacturer-reported intra-assay and inter-assay coefficients of variation were 4.50–6.18% and 7.76–8.55%, respectively, for PON1; 4.53–6.10% and 6.03–8.18% for TNF-α; 5.06–5.96% and 6.06–8.49% for IL-6; 4.68–5.54% and 6.31–8.42% for NT-proBNP; and <10% for both in-tra-assay and inter-assay variability for 3-nitrotyrosine. All assays were performed according to the manufacturer’s instructions. Absorbance measurements and automated plate washing were carried out using an 800 TS ELISA microplate reader and a Biotek 50 TS microplate washer (Agilent Technologies Inc., Santa Clara, CA, USA).

### 4.4. Echocardiographic Evaluation

All participants underwent comprehensive transthoracic echocardiography performed by an experienced cardiologist using a commercially available ultrasound system. The following parameters were assessed: left atrial diameter, right atrial diameter, right ventricular diameter, tricuspid annular plane systolic excursion (TAPSE), and pulmonary artery systolic pressure (PSAP). Measurements were obtained according to the recommendations of the American Society of Echocardiography and the European Association of Cardiovascular Imaging.

### 4.5. Statistical Analysis

Statistical analyses were performed using IBM SPSS Statistics version 20 (IBM Corp., Armonk, NY, USA). The distribution of continuous variables was assessed using the Shapiro–Wilk test. Continuous variables are presented as median and interquartile range (IQR), whereas categorical variables are presented as absolute numbers and percentages. Comparisons among OSA severity groups were performed using the Kruskal–Wallis test. Comparisons between two groups were performed using the Mann–Whitney U test. Categorical variables were compared using the chi-square test or Fisher’s exact test, as appropriate. Associations between continuous variables were assessed using Spearman’s rank correlation coefficient. To investigate the relationship between oxidative stress biomarkers and systemic inflammatory activity, univariate and multivariable linear regression analyses were performed using IL-6 as the dependent variable. Multivariable models were adjusted for age, body mass index, and diabetes mellitus, selected a priori based on their potential influence on inflammatory status. A two-sided *p*-value < 0.05 was considered statistically significant.

### 4.6. Ethical Considerations

The study protocol was approved by the Ethics Committee of “Iuliu Hațieganu” University of Medicine and Pharmacy Cluj-Napoca (Approval No. 119, 17 May 2022). All participants provided written informed consent prior to enrollment. The study was conducted in accordance with the principles of the Declaration of Helsinki.

## 5. Conclusions

In this cohort of patients with obstructive sleep apnea syndrome, conventional OSA severity categories were not associated with significant differences in oxidative stress biomarkers, inflammatory markers, or echocardiographic parameters. Diabetes mellitus was associated with modest differences in atrial dimensions and pulmonary artery systolic pressure, suggesting a potential contribution of cardiometabolic comorbidity to cardiovascular remodeling in OSA. Oxidative stress biomarkers were strongly interrelated and showed significant associations with IL-6 concentrations. Furthermore, MDA and OSI remained significantly associated with IL-6 after adjustment for age, body mass index, and diabetes mellitus. These findings highlight a potential link between oxidative stress and systemic inflammation in patients with OSA, independent of age, body mass index, and diabetes mellitus, and support further investigation of oxidative stress pathways as biomarkers of disease-related biological activity.

## Figures and Tables

**Table 1:** Baseline demographic, clinical, echocardiographic, oxidative stress, and inflammatory characteristics of the study population.

Variable	Total Cohort (*n* = 72)
**Demographic and clinical characteristics**	
Age (years)	59 (49–64)
Male sex, *n* (%)	53 (73.6)
BMI (kg/m^2^)	34.2 (31.3–38.0)
Smoking, *n* (%)	31 (43.1)
Hypertension, *n* (%)	67 (93.1)
Diabetes mellitus, *n* (%)	16 (22.2)
Ischemic heart disease, *n* (%)	20 (27.8)
Chronic pulmonary disease, *n* (%)	25 (34.7)
Atrial fibrillation, *n* (%)	2 (2.8)
eGFR (mL/min/1.73 m^2^)	96.2 (84.7–103.4)
**Sleep study parameters**	
AHI	27.6 (16.4–57.9)
ODI	27.6 (15.9–58)
**Echocardiographic parameters**	
LA diameter (mm)	30 (28–31.5)
RA diameter (mm)	17 (16–18)
RV diameter (mm)	24 (23–24)
TAPSE (mm)	26 (24–29)
PSAP (mmHg)	32 (25.5–36)
**Inflammatory and cardiac biomarkers**	
CRP (mg/L)	3.04 (1.64–5.07)
TNF-α (pg/mL)	52.9 (34.6–78.9)
IL-6 (pg/mL)	3.18 (1.95–6.01)
**Cardiac biomarker**	
NT-proBNP (ng/mL)	0.184 (0.121–0.251)
**Oxidative stress biomarkers**	
MDA (µmol/L)	4.42 (3.68–5.17)
TOS (µmol H_2_O_2_ equiv./L)	125.1 (65.6–155.9)
TAC (mmol Trolox equiv./L)	1.15 (1.14–1.16)
NO (µmol/L)	67.2 (57.0–82.0)
OSI	108.7 (57.3–134.0)
PON1 (U/L)	4.00 (3.04–5.66)
3-NT (ng/mL)	20.64 (6.07–36.41)

Data are presented as median (interquartile range [IQR]) for continuous variables and number (percentage) for categorical variables. Abbreviations: BMI, body mass index; eGFR, estimated glomerular filtration rate; AHI, apnea-hypopnea index; ODI, oxygen desaturation index; CRP, C-reactive protein; LA, left atrial diameter; RA, right atrial diameter; RV, right ventricular diameter; TAPSE, tricuspid annular plane systolic excursion; PSAP, pulmonary artery systolic pressure; MDA, malondialdehyde; TOS, total oxidant status; TAC, total antioxidant capacity; NO, nitric oxide; OSI, oxidative stress index; PON1, paraoxonase-1; 3-NT, 3-nitrotyrosine; TNF-α, tumor necrosis factor-alpha; IL-6, interleukin-6; NT-proBNP, N-terminal pro-B-type natriuretic peptide.

**Table 2:** Clinical, echocardiographic, oxidative stress, and inflammatory parameters according to OSA severity.

	Mild OSA (*n* = 15)	Moderate OSA (*n* = 19)	Severe OSA (*n* = 38)	*p*-Value
Age (years)	59 (46–62)	61 (52–71)	54 (46.5–62)	0.226
BMI (kg/m^2^)	32.4 (29.3–38.0)	33.5 (30.1–38.5)	35.1 (32.1–38.2)	0.507
eGFR (mL/min/1.73 m^2^)	98.2 (81.2–104.0)	94.5 (87.1–102.8)	96.2 (84.7–105.3)	0.891
CRP (mg/L)	2.81 (1.44–5.54)	3.28 (1.76–6.78)	2.70 (1.54–4.56)	0.513
LA diameter (mm)	30 (29–32)	30 (29–30)	29 (28–32)	0.698
RA diameter (mm)	17 (16–17)	17 (17–18)	17 (16–18)	0.086
RV diameter (mm)	24 (23–24)	24 (24–24)	24 (23–24)	0.324
TAPSE (mm)	28 (24–29)	28 (21–29)	26 (24–28)	0.536
PSAP (mmHg)	32 (28–35)	32 (18–35.5)	34 (26–36)	0.416
MDA (µmol/L)	4.42 (3.61–5.02)	4.29 (3.65–5.02)	4.52 (3.85–5.38)	0.608
TOS (µmol H_2_O_2_ equiv./L)	98.4 (65.6–154.1)	106.5 (46.4–143.8)	132.7 (77.8–160.6)	0.294
TAC (mmol Trolox equiv./L)	1.148 (1.137–1.158)	1.146 (1.139–1.157)	1.151 (1.145–1.168)	0.326
NO (µmol/L)	64.8 (53.9–75.5)	62.6 (56.1–74.0)	67.6 (61.4–85.3)	0.326
OSI	85.2 (57.3–130.6)	93.5 (40.9–125.8)	116.2 (68.6–137.7)	0.299
PON1 (U/L)	4.00 (3.58–5.39)	3.34 (2.91–5.14)	4.12 (2.91–5.66)	0.497
3-NT (ng/mL)	24.0 (6.52–38.1)	13.6 (1.96–28.8)	28.4 (8.40–39.3)	0.205
TNF-α (pg/mL)	71.2 (45.7–88.5)	41.9 (27.6–62.2)	53.2 (33.1–82.6)	0.333
IL-6 (pg/mL)	3.01 (1.79–5.72)	3.98 (2.31–5.29)	3.10 (1.99–7.10)	0.788
NT-proBNP (ng/mL)	0.174 (0.075–0.232)	0.167 (0.141–0.236)	0.200 (0.110–0.251)	0.653

Data are presented as median (interquartile range [IQR]). Comparisons among groups were performed using the Kruskal–Wallis test. Abbreviations: OSA, obstructive sleep apnea; BMI, body mass index; eGFR, estimated glomerular filtration rate; CRP, C-reactive protein; LA, left atrial diameter; RA, right atrial diameter; RV, right ventricular diameter; TAPSE, tricuspid annular plane systolic excursion; PSAP, pulmonary artery systolic pressure; MDA, malondialdehyde; TOS, total oxidant status; TAC, total antioxidant capacity; NO, nitric oxide; OSI, oxidative stress index; PON1, paraoxonase-1; 3-NT, 3-nitrotyrosine; TNF-α, tumor necrosis factor-alpha; IL-6, interleukin-6; NT-proBNP, N-terminal pro-B-type natriuretic peptide.

**Table 3:** Comparison of echocardiographic, oxidative stress, and inflammatory parameters according to diabetes mellitus status.

Variable	No Diabetes (*n* = 56)	Diabetes (*n* = 16)	*p*-Value
LA diameter (mm)	30 (28–30)	31 (29–33.8)	0.041
RA diameter (mm)	17.0 (16–18)	17.5 (17–18)	0.010
PSAP (mmHg)	32 (20–36)	35 (32–37)	0.026
MDA (µmol/L)	4.29 (3.61–5.20)	4.78 (3.99–5.04)	0.461
TOS (µmol H_2_O_2_ equiv./L)	107.3 (49.0–155.4)	142.3 (110.0–161.2)	0.082
OSI	94.2 (43.2–133.8)	123.7 (96.0–138.5)	0.078

Data are presented as median (interquartile range [IQR]). Comparisons between groups were performed using the Mann–Whitney U test. Abbreviations: LA, left atrial diameter; RA, right atrial diameter; PSAP, pulmonary artery systolic pressure; MDA, malondialdehyde; TOS, total oxidant status; OSI, oxidative stress index.

**Table 4:** Spearman correlation matrix of oxidative stress, nitrosative stress, inflammatory, and cardiac biomarkers.

Variable	MDA	TOS	OSI	IL-6	TNF-α	3-NT	CRP	NT-proBNP	PON1
**MDA**	—	0.752 ***	0.740 ***	0.364 **	−0.202	0.305 *	0.047	−0.005	−0.092
**TOS**	0.752 ***	—	0.999 ***	0.259 *	−0.223	0.375 **	0.008	0.144	−0.061
**OSI**	0.740 ***	0.999 ***	—	0.257 *	−0.227	0.374 **	0.005	0.147	−0.059
**IL-6**	0.364 **	0.259 *	0.257 *	—	−0.007	−0.030	0.084	0.232	−0.058
**TNF-α**	−0.202	−0.223	−0.227	−0.007	—	−0.046	−0.119	−0.030	0.124
**3-NT**	0.305 *	0.375 **	0.374 **	−0.030	−0.046	—	−0.016	0.084	−0.090
**CRP**	0.047	0.008	0.005	0.084	−0.119	−0.016	—	0.105	−0.021
**NT-proBNP**	−0.005	0.144	0.147	0.232	−0.030	0.084	0.105	—	−0.087
**PON1**	−0.092	−0.061	−0.059	−0.058	0.124	−0.090	−0.021	−0.087	—

Data presented as Spearman’s correlation coefficients (ρ). * *p* < 0.05; ** *p* < 0.01; *** *p* < 0.001. Abbreviations: MDA, malondialdehyde; TOS, total oxidant status; OSI, oxidative stress index; IL-6, interleukin-6; TNF-α, tumor necrosis factor-alpha; 3-NT, 3-nitrotyrosine; CRP, C-reactive protein; NT-proBNP, N-terminal pro-B-type natriuretic peptide; PON1, paraoxonase-1.

**Table 5:** Univariate and Multivariable Linear Regression Analyses for IL-6 Concentrations.

Predictor	B	95% CI for B	Standardized β	*p*-Value
**Univariate model (MDA)**				
MDA (µmol/L)	1.562	0.034 to 3.090	0.238	0.045
**Model statistics**			R^2^ = 0.057	
**Multivariable model 1**				
Age	0.080	−0.091 to 0.251	0.121	0.354
BMI	0.023	−0.285 to 0.332	0.018	0.880
Diabetes mellitus	2.230	−1.847 to 6.308	0.136	0.279
MDA (µmol/L)	1.776	0.110 to 3.442	0.271	0.037
**Model statistics**			R^2^ = 0.098; Adjusted R^2^ = 0.044	*p* = 0.140
**Univariate model (OSI)**				
OSI	0.042	0.006 to 0.079	0.272	0.022
**Model statistics**			R^2^ = 0.074	
**Multivariable model 2**				
Age	0.074	−0.095 to 0.243	0.112	0.384
BMI	−0.016	−0.321 to 0.289	−0.013	0.917
Diabetes mellitus	1.604	−2.615 to 5.822	0.098	0.451
OSI	0.043	0.003 to 0.083	0.277	0.034
**Model statistics**			R^2^ = 0.100; Adjusted R^2^ = 0.046	*p* = 0.131

Multivariable models were adjusted for age, body mass index (BMI), and diabetes mellitus. B = unstandardized regression coefficient; β = standardized regression coefficient; CI = confidence interval.

## Data Availability

The original contributions presented in the study are included in the current article, and further inquiries can be directed to the first author.

## Abbreviations

The following abbreviations are used in this manuscript:

3-NT	Nitrosative stress marker 3-nitrotyrosine
AHI	Apnea–hypopnea index
BMI	Body mass index
CI	Confidence interval
CRP	C-reactive protein
eGFR	Estimated glomerular filtration rate
ELISA	Enzyme-Linked Immunosorbent Assay
IL-6	Interleukin-6
IQR	Interquartile range
LA	Left atrial diameter
MDA	Malondialdehyde
NF-κB	Nuclear factor-kappa B
NO	Nitric oxide
NT-proBNP	N-terminal pro-B-type natriuretic peptide
OSA	Obstructive sleep apnea
OSI	Oxidative stress index
PON1	Paraoxonase-1
PSAP	Pulmonary artery systolic pressure
RA	Right atrial diameter
ROS	Reactive oxygen species
RV	Right ventricular diameter
TAC	Total antioxidant capacity
TAPSE	Tricuspid annular plane systolic excursion
TNF-α	Tumor necrosis factor-alpha
TOS	Total oxidant status
